# Correlation of hematological indices and ratios derived from them with FIQR scores in fibromyalgia

**DOI:** 10.12669/pjms.345.15169

**Published:** 2018

**Authors:** Marwan Salih Mohammud Al-Nimer, Talar Ahmad Merza Mohammad

**Affiliations:** 1 Dr. Marwan Salih Mohammud Al-Nimer, MD, PhD. Department of Pharmacology and Toxicology, Clinical Pharmacy, Hawler Medical University, Erbil, Iraq; 2 Dr. Talar Ahmad Merza Mohammad, MSc. Department of Pharmacology and Toxicology, Clinical Pharmacy, Hawler Medical University, Erbil, Iraq

**Keywords:** Fibromyalgia, Hematological indices, Diagnosis, Prediction

## Abstract

**Objectives:**

To determine the hematological indices and ratios derived from them in patients with fibromyalgia and to correlate the scores of Fibromyalgia Impact Questionnaire Revised (FIQR) with the ratios.

**Methods:**

This case control study was performed in the College of Pharmacy at Hawler Medical University in Erbil-Iraq, from November 2016 to June 2017, and it included 40 healthy subjects (Group I) and 150 newly diagnosed FM (Group II). The American College of Rheumatology -10 (ACR-10) diagnostic criteria were used in the diagnosis of FM. The scores of the Revised Fibromyalgia Questionnaire Impact (FIQR), and tender points were calculated, and the hematological indices and ratios were determined.

**Results:**

Group II showed significantly higher mean values of hematological indices and the ratios of neutrophil to lymphocyte (NLR), derived neutrophil to lymphocyte (dNLR) and platelet to lymphocyte (PLR). Group II patients have a significant higher score of FIQR. A significant correlation between the total score of FIQR with the hematological ratios (F=4.143, R=0.355, R^2^=0.126, p=0.002) with a variability of 12.6%.

**Conclusion:**

We conclude that the hematological indices are significantly altered and they are significantly correlated with the total score of fibromyalgia impact questionnaire revised.

## INTRODUCTION

Fibromyalgia is a chronic non-articular disease diagnosed clinically because there are no specific analytical diagnostic criteria. There is an assumption that chronic inflammatory process may play a role in the pathogenesis of Fibromyalgia (FM). Backryd and colleagues found increase of certain inflammatory protein levels in the cerebrospinal fluid and plasma in patients with FM suggesting systemic and neuroinflammation.^1^ Circulating monocytes as a marker of inflammation was assessed in FM patients, and it has been found that the total percentages of circulating monocytes subpopulation not significantly differed between FM patients and healthy subjects.^2^ Long standing fibromyalgia patients have increased plasma levels of cytokines including, interleukins (1L-1β, IL-4,IL-5, IL-6, IL-12, and IL-15), interferon (IFN)-α, and Tumor Necrosis Factor (TNF)-α.^3^ A study has documented that there is no significant fluctuation in the serum levels of proinflammatory cytokines including, IL-6, IL-8, and TNF-α while the anti-inflammatory IL-10 level significantly increased.^4^

Chronic widespread pain conditions, including fibromyalgia characterized by increase plasma levels of the endogenous lipids that act as anti-inflammatory mediators without alterations in the plasma levels of pro-inflammatory cytokines.^5^

Fibromyalgia patients presented with cardiovascular events have a significant high value of Mean Platelet Volume (MPV) (mean±SD: 8.09±0.84 fL) compared with the corresponding value of healthy subjects (mean±SD:7.73±0.65 fL).^6^

Neutrophil to Lymphocyte Ratio (NLR), platelet distribution width (PDW) and MPV has been associated as a systemic inflammatory marker.^7^ They found that NLR and MPV were significantly higher in FM patients and they suggested that PDW is a predicting marker of FM.^7^ Previous studies suggested to calculate the platelet to lymphocyte ratio as a marker of myocardial ischemia^8^; derived Neutrophil to Lymphocyte Ratio (dNLR) as a marker of survival in debilitating diseases^9^; and monocyte to High Density Lipoprotein-cholesterol (HDL-c) ratio as a marker of low-grade inflammation and metabolic syndrome.^10^ The rationale of this study is that the inflammatory marker panel of rheumatic diseases is usually negative in FM patients, but there are changes in the markers that are related to inflammation including proteins, lipids, and even the hematological indices in FM. Therefore, it is worth trying to measure the hematological indices, and high density lipoprotein as well as their ratios as indicators of inflammation in newly diagnosed FM patients; and to relate these values to the scores of the clinical presenting symptoms of FM.

## METHODS

This case control study was conducted in the Department of Pharmacology and Toxicology-Clinical Pharmacy in the College of Pharmacy at Hawler Medical University in Erbil-Iraq, between November 2016 and June 2017. The Institutional Ethics Review Committee approved this study under the Helsinki Declaration. Each participant in this study signed informed consent.

Eligible patients were aged > 18-years of both genders. The patients were referred by the consultant rheumatologists working in the Teaching hospitals, Consultant Public clinics, and the private clinics. The criteria of inclusions are patients presented with recent onset of widespread pain suggesting FM of more than three months duration; negative rheumatic laboratory profile; and met the American College of Rheumatology (ACR)-10 classification criteria for FM.^11^,^12^ Criteria of exclusions are; chronic inflammatory disease, clinical or laboratory evidence of acute or subacute infections, diabetes mellitus, hypertension, hypothyroidism, dyslipidemia, psychiatric or neurological disorder, hematological disorder, pregnancy, lactate nursing, and using non-steroidal and steroidal anti-inflammatory drugs.

The sample size of two independent samples was calculated after doing the pilot study on the healthy subjects and fibromyalgia. The mean, standard deviations, and the difference between the means were calculated from the pilot study. The power of the study 1-β (where the β is type II error) is fixed at 80% (0.8) and the significance level (α), which is the type I error, is fixed at 5% (≤0.05). Then the following equation was used to calculate the sample size:

Sample size = 1 + 2C (Standard deviation/difference between means)^2^, where C represents the Constant value that derived from the statistical tables and it equals to 7.85 when the 1-β =0.8 and α=0.05. Because fibromyalgia is more common in women than men. We recruited a sample size of healthy subjects in respect to the gender (men to women ratio) which is approximately equal to that of FM patients.

The participants were grouped in to Group I comprised of 40 healthy volunteers (35 women and 5 men) and Group II comprised of 150 FM patients (130 women and 20 men).

The researchers interviewed each participant, reported their characteristics data, examined the tender points, and scoring the Revised Fibromyalgia Impact Questionnaire (FIQR).^13^-^15^

A venous blood sample (5 ml) was obtained from each participant from the antecubital region in the morning after overnight fasting. Venous blood samples collected in the tubes with and without an anticoagulant (EDTA). The hematological indices of the red blood cell, white blood cell, and blood platelet were electronically recorded by using a hematology analyzer (Beckman Coulter Inc., USA). In addition, we calculate neutrophil to lymphocyte ratio (NLR); derived Neutrophil to Lymphocyte Ratio (dNLR); Platelet to Lymphocyte Ratio (PLR); and Lymphocyte to Monocyte Ratio (LMR). The dNLR was calculated as the absolute count of neutrophil divided by the absolute white cell count of leukocytes minus the absolute count of neutrophils.^16^

Sera of the venous blood without anti-coagulant tubes separated by centrifugation at 3000 rpm, for 10 minutes for determination of High Density Lipoprotein-cholesterol (HDL-c) by using a colorimetric enzymatic reaction kit. The monocyte to HDL-c ratio was calculated.

### Statistical Analysis

Statistical analyses were performed using SPSS version 20.0 for Windows. The results were expressed as mean ± SD. Two-tailed, unpaired student’s t-test was used to compare the difference for continuous data, and the Chi-squared test for category data. The difference considered statistically significant when p ≤ 0.05. Receiving operating characteristic analysis applied to estimate the cutoff values of sensitivity and specificity of the hematological indices, and the multi-variables linear regression test used to assess the correlations between these indices and to calculate the prediction percentage.

## RESULTS

There is a non-significant difference between Group I and Group II in the characteristics of the participants, including the mean age and the frequency (percentage) of residency, smoking and positive family history of FM (Table-I).

**Table-I:** Characteristics of participants included in the study.

Variables	Group I (n=40)	Group II (n=150)	P value
Gender (F:M) Ratio	35:5 (7:1)	130:20 (6.5:1)	0.890
Age (year)	42.4±7.4	41.6±7.6	0.553
***Residency***			
Urban	32 (80)	110 (73.3)	0.389
Rural	8 (20)	40 (26.7)	
Family history of Fibromyalgia	3 (7.5)	21 (14)	0.261
***Smoking***			
Smoker	7 (17.5)	22 (14.7)	
Ex-smoker	5 (12.5)	14 (9.3)	0.728
No smoker	28 (70)	114 (76)	

The results are expressed as mean ± SD and number (percentage). *P* value is calculated by using unpaired two tailed Student ’t’ test and Chi-squared test for category data between Group I (healthy subjects) and Group II (fibromyalgia patient). NC: not calculated because of small number.

**Fig.1:**
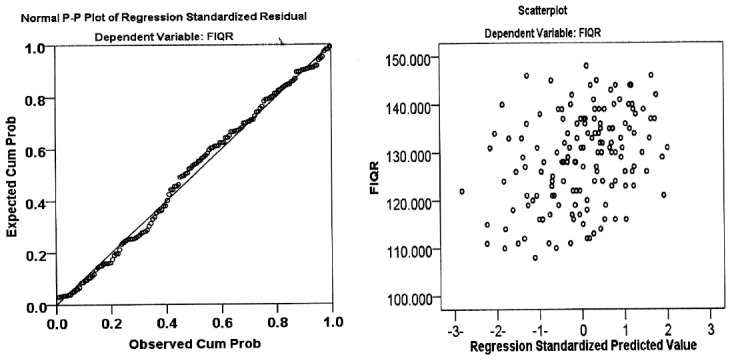
Multivariable linear regression plot taking the score of fibromyalgia impact questionnaire (FIQR) as dependent variable and neutrophil to lymphocyte ratio; derived neutrophil to lymphocyte ratio; platelet to lymphocyte ratio; lymphocyte to monocyte ratio; and monocyte to high density lipoprotein ratio as independent variables. (F=4.143, R=0.355, R^2^=0.126, p=0.002).

The results of hematological indices showed that Group II patients have significant high RDW, lymphocyte (percentage), monocytes (percentage) MPV, and PDW while the neutrophil (percentage) significantly reduced (Table-IIa). The mean values of NLR and dNLR are significantly reduced in Group II compared with Group I while non-significant differences in the PLR, LMR, and MHR observed (Table-IIb). The areas under the curve of the NLR, dNLR, PLR, and LMR values are higher than 0.5, while the MHR value is less than 0.5 in Group II as compared with Group I (Table-III). Group II patients had a significantly high score of FIQR domains and tender points (Table-IV). Multivariable linear regression analysis showed a significant correlation between total score of FIQR as dependent variable with NLR, dNLR, PLR, LMR and MHR as independent predictors (F=4.143, R=0.355, p=0.002) with a variety of 12.6% (R^2^=0.126) (Fig.1).

**Table IIa:** Calculated hematological indices as proinflammatory and cardiovascular risk markers in participants included in the study.

Variables	Group I (n=40)	Group II (n=150)	P value
RBC count ⊆10^6^ (mm^3^)	4.829±0.330	4.742±0.410	0.217
Hemoglobin (g/dl))	12.92±1.40	12.77±1.44	0.557
Hematocrit (%)	42.79±4.13	41.58±4.59	0.132
Mean cell hemoglobin (pg/cell)	26.79±2.54	27.16±2.76	0.445
Mean cell hemoglobin concentration (g/dl)	30.29±2.80	30.82±2.24	0.210
Mean cell volume (fL)	88.87±9.04	87.62±10.55	0.421
RDW (%)	11.85±0.65	12.84±0.84	<0.001
WBC count ⊆10^3^ (mm^3^)	7.728±0.987	7.705±1.016	0.920
Neutrophil (%)	61.7±1.9	60.5±2.4	0.004
Lymphocyte (%)	29.3±1.9	30.5±2.3	0.003
Monocyte (%)	5.94±0.47	6.28±0.62	0.002
Eosinophil (%)	1.62±0.43	1.58±0.33	0.525
Basophil (%)	1.43±0.64	1.17±0.74	0.044
Platelet count Í10^3^ (mm^3^)	279.1±52.4	289.7±54.5	0.272
Plateletcrit (%)	0.171±0.02	0.181±0.036	0.093
Mean platelet volume (fL)	8.71±0.89	10.50±1.67	<0.001
Platelet distribution width (%)	14.9±1.2	16.3±1.4	<0.001

The results are expressed as mean ± SD and number. P value is calculated by using unpaired two tailed Student- t test between Group I (healthy subjects) and Group II (fibromyalgia patients).

**Table- IIb:** Calculated hematological indices as proinflammatory and cardiovascular risk markers in participants included in the study.

Variables	Group I (n=40)	Group II (n=150)	P value
Neutrophil-lymphocyte ratio	2.119±0.207	1.998±0.236	0.0036
Derived neutrophil-lymphocyte ratio	1.618±0.134	1.539±0.160	0.0046
Platelet-lymphocyte ratio	125.11±29.37	126.37±41.9	0.858
Lymphocyte-monocyte ratio	4.961±0.533	4.910±0.633	0.641
Monocyte-high density lipoprotein- cholesterol ratio	0.994±0.172	1.009±0.212	0.680

The results are expressed as mean ± SD and number. P value is calculated by using unpaired two tailed Student-t test between Group I (healthy subjects) and Group II (fibromyalgia patients).

**Table-III:** Clinical assessment of participants using Revised Fibromyalgia Impact Questionnaire and tender points testing.

Variables	Group I (n=40)	Group II (n=150)	P value
Duration of symptoms (weeks)		19.76±2.59	
Scoring of FIQR			
Function	23.8±3.1	52.4±4.9	<0.001
Global impact	5.0±1.6	13.4±1.2	<0.001
Symptoms	24.6±3.3	62.7±5.2	<0.001
Total	53.4±5.9	128.5±8.6	<0.001
Tender points (Number)	4.8±1.4	13.9±1.6	<0.001

The results are expressed as mean ± SD and number. P value is calculated by using unpaired two tailed Student- t test between Group I (healthy subjects), Group II (fibromyalgia patients),

FIQR: revised fibromyalgia impact questionnaire.

**Table IV:** The area under the curve of the hematological indices as discriminators of diagnosis of fibromyalgia.

Hematological ratios	Group I (n=40)		Group II (n=150)	

	Area under curve	95% confidence interval	Area under curve	95% confidence interval
Neutrophil to lymphocyte	0.319	0.235-0.403	0.681	0.597-0.765
Derived neutrophil to lymphocyte	0.334	0.247-0.421	0.666	0.579-0.753
Platelet to lymphocyte	0.468	0.368-0.576	0.532	0.433-0.632
Lymphocyte to monocyte	0.472	0.376-0.567	0.528	0.433-0.624
Monocyte to high density lipoprotein	0.519	0.423-0.615	0.481	0.385-0.577

Null hypothesis: true area=0.5.

## DISCUSSION

The results of this study show that the hematological indices as proinflammatory markers are significantly altered in FM patients and therefore can serve as predictors of disease severity as they show significant correlation with FIQR scores. This study provides new inflammatory markers without wide variation. In addition, there is no significant difference between Group I and II in the characteristic features of the participants, which eliminate the bias in the present study. A significant increase of the RDW percentage in Group II suggested evidence of existing inflammatory process as one study showed an association between inflammation and the RDW.^17^

Significantly low levels of neutrophil and basophil and high levels of lymphocyte levels among FM patients strongly indicated that there is an associated inflammation. Bote and colleagues have found that the neutrophil function (e.g., chemotaxis, phagocytosis and fungicidal capacity) increased in FM, and they suggested that this test is a useful investigation for assessment the anti-inflammatory effect of the non-pharmacological management of FM.^18^ A recent study included twenty FM patients and showed that there is a significant role of the monocytes in mediating the pain.^2^ The platelet activity is increased in FM indicating there is activity of inflammation, and the patients are at risk of coronary artery disease. The results of this study are in agreement with Haliloğlu 6 who found significant high mean platelet volume and suggested that the patients are at risk of cardiovascular disease. A significantly high value of platelet width distribution percentage that reported in this study indicates that there is an active inflammatory process in patients and they are at risk of cardiovascular disease.^19^ The significant low value of NLR that observed in Group II is simply due to the difference between the mean values of neutrophil and lymphocytes were -1.2% and + 0.34% respectively. A recent study suggested an optimal value of NLR, and PLR ratios are 2.72 and 132.88 as good markers to discriminate inflammatory bowel disease from other conditions.^20^ Therefore, the NLR could be a good marker to discriminate fibromyalgia from other painful conditions. It is well known that dNLR is a prognostic marker of malignancies (an optimal cutoff value is 1.73), and the significant low mean value of dNLR at the time of diagnosis of fibromyalgia indicating that the disease is a benign condition.^9^ The area under the curve shows that NLR and dNLR are good discriminators to diagnose FM. The ratios of hematological indices are significantly correlated with the FIQR scores at the time of the diagnosis of FM and prediction of the number of the tender points can be derived from the hematological indices in 12.6% of our patients. We have demonstrated a higher red distribution width value while other studies showed a low value of red distribution width.^7^ Moreover, our findings regarding the value of mean platelet volume is in agreement with other.^6^,^7^

Our results indicate that we can predict the severity and the future prognosis of FM by measuring the hematological indices. Therefore, determination of the cutoff values of these hematological indices and their ratios in large sample size can serve as a useful analytic test for diagnosis and prognosis of FM.

## CONCLUSION

The hematological indices are significantly altered and they are significantly correlated with the total score of fibromyalgia impact questionnaire revised. Since correlation of hematological ratios with the scores of FIQR is found to be significant therefore these indices and ratios could serve as a useful marker for diagnosis and prognosis of fibromyalgia. However multi-centric studies with large sample size should be conducted to determine the cut off values of these hematological indices and ratios in patients with fibromyalgia.
